# Tunable emission from H-type supramolecular polymers in optical nanocavities†

**DOI:** 10.1039/d3cc05877h

**Published:** 2024-02-09

**Authors:** Giulia Lavarda, Anton M. Berghuis, Kripa Joseph, Joost J. B. van der Tol, Shunsuke Murai, Jaime Gómez Rivas, E. W. Meijer

**Affiliations:** a Institute for Complex Molecular Systems and Laboratory of Macromolecular and Organic Chemistry, Eindhoven University of Technology Eindhoven 5600 MB The Netherlands g.lavarda@tue.nl e.w.meijer@tue.nl; b Eindhoven Hendrik Casimir Institute and Institute for Complex Molecular Systems, Eindhoven University of Technology Eindhoven 5600 MB The Netherlands; c Department of Material Chemistry, Graduate School of Engineering, Kyoto University Katsura, Nishikyo Kyoto 6158510 Japan

## Abstract

H-type supramolecular polymers with preferred helicity and highly efficient emission have been prepared from the self-assembly of chiral tetraphenylene-based monomers. Implementation of the one-dimensional fibers into dielectric nanoparticle arrays allows for a significant reshaping of fluorescence due to weak light–matter coupling.

## Abstract 2

Tunable emission from H-type supramolecular polymers prepared by self-assembly of tetraphenylethylene-based monomers is achieved by harnessing the surface lattice resonances of dielectric TiO_2_ nanoparticles.

## Graphical abstract

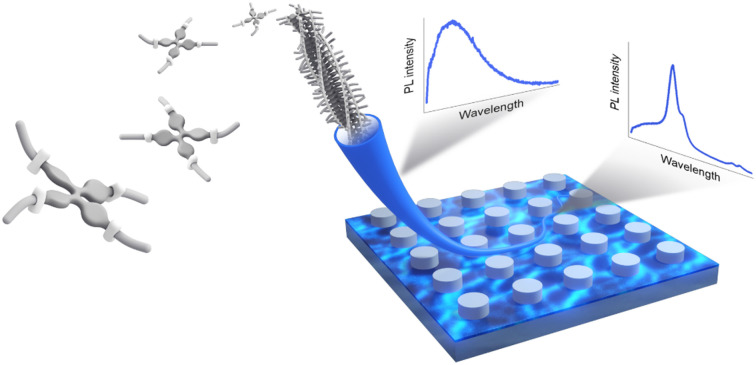

Tunable emission in optically active materials is a desirable feature for a wide range of applications, from optoelectronics to biomedicine.^1–4^ Due to their structural and electronic properties, π-conjugated chromophores are ideal building blocks for the preparation of functional materials for optical technologies.^5,6^ By exploiting the supramolecular interactions between π-surfaces, the molecular arrangement and aggregate morphology can be precisely controlled at the microscopic scale.^7^ Nevertheless, drastic quenching of fluorescence is often observed in stacked architectures of emissive chromophores, limiting optical performance.

The proper molecular design of organic building blocks provides however an effective strategy for the preparation of luminescent assemblies. Recently, this phenomenon is often referred to as aggregation-induced emission (AIE), but it has been known for much longer.^8,9^ The emission in these cases results from the suppression of non-radiative deactivation pathways through the restriction of intramolecular rotational or vibrational modes in the aggregate state, with tetraphenylethylene (TPE) serving as a prototypical example.^10^ The optical properties of these emissive materials make them interesting candidates for implementation in light-emitting, sensing and imaging technologies, among others.^8,11–13^

Therefore, the study of fluorescent H-type aggregates – where molecular units are stacked in a face-to-face arrangement – is critical to the advancement of emission-based applications. In this context, long-range ordered supramolecular polymers can be conveniently used as model systems to gain insights into the fundamental effects underlying optoelectronic materials.^14,15^ Despite numerous reports on TPE aggregates, this core has rarely been employed in the preparation of H-type one-dimensional supramolecular polymers.^16–19^ Instead, *C*_3_-symmetric discotics decorated with amide motifs are typically used for the latter, ensuring the formation of helical fibers.^20^

On the other hand, light–matter coupling has attracted much attention in the last decades as a tool to engineer the optical properties of materials, showing significant potential for improving the performance of organic electronics.^21,22^ In the weak coupling regime, excitonic transitions exchange energy with resonant photonic modes, resulting in the modification of emission properties.^23,24^ Although this research area is rapidly expanding, the effect of light–matter coupling on the optical properties of H-type supramolecular polymers received little attention.^25^

In this communication, we report the tuning of emission from one-dimensional H-type assemblies *via* weak coupling to the surface lattice resonances in arrays of dielectric TiO_2_ nanoparticles. To induce self-assembly into fluorescent supramolecular polymers, we designed a monomer featuring a TPE core decorated at the *para*-position of the phenyl rings with amide functionalities and solubilizing (*S*)-3,7-dimethyloctyl chains (*S*-TPE, Fig. 1). The target compound was synthesized by an amidation reaction between tetrakis(4-carboxy)-tetraphenylethylene precursor and 3,7-dimethyloctylamine using COMU as the coupling reagent. The product was purified by conventional laboratory techniques and fully characterized by ^1^H-NMR, ^13^C-NMR and mass spectrometry (see ESI† for further details).

**Fig. 1:**
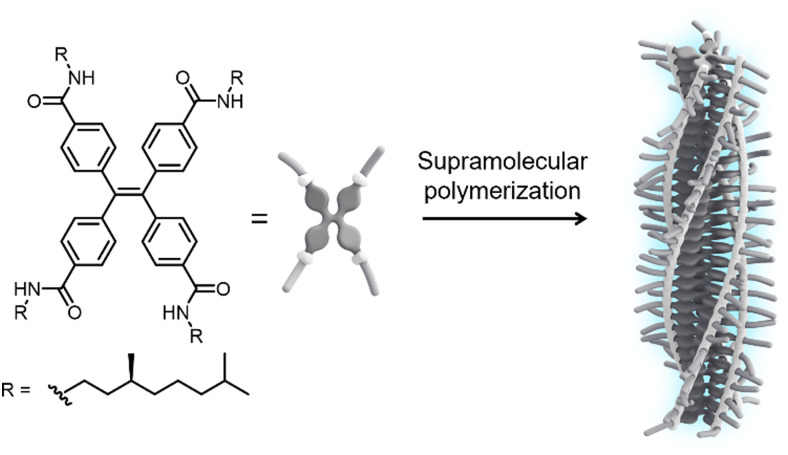
Chemical structure of the *S*-TPE monomer and schematic representation of its assembly into emissive H-type supramolecular polymers with preferred helicity.

UV-vis measurements of *S*-TPE solutions in 1,1,2,2-tetrachloroethane (TCE) show the typical absorption profile of TPE chromophores, with maxima at 333 and 296 nm (Fig. S6a, ESI†). The linear dependence of absorbance on concentration and the absence of any Cotton effect indicate that the compound is monomerically dissolved at room temperature in the concentration range studied (namely 5–200 μM, Fig. S6b and d, ESI†). The absence of significant emission under these experimental conditions is attributed to the activation of competitive decay pathways resulting from the free rotation of the phenyl moieties in the monomer (Fig. S6c, ESI†). In this context, the presence of a weak emission around 500 nm at concentrations higher than 30 μM can be attributed to the formation of excimer species.^26^

Next, the ability of *S*-TPE to assemble in decalin/TCE mixtures under dilute conditions (*i.e.*, 20 μM) was investigated. The addition of apolar solvent to polar TCE resulted in the appearance and gradual increase of a CD signal in the 260–400 nm region for decalin ratios higher than 85%, with a minimum at 322 nm and a maximum at 270 nm (Fig. S7a, ESI†). At the same time, a marked improvement in emission is observed with increasing amounts of alkane solvent (Fig. S7b, ESI†).

Variable temperature (VT) UV-vis, CD and emission spectroscopy studies were performed on 20 μM solutions in decalin/TCE 9 : 1 (v/v) to further investigate the self-assembly behavior of *S*-TPE. While the absence of any CD signal at 100 °C indicates that *S*-TPE is present in solution as a monomeric species, the appearance and successive enhancement of a Cotton effect upon controlled cooling to 20 °C at a rate of 1 °C min^−1^ indicates an ongoing self-assembly process (Fig. 2a). These observations are consistent with previous reports on stacked aggregates of chiral TPE species, indicating the formation of H-type supramolecular polymers with a preferred helicity.^18^ In parallel, spectroscopic changes are observed in the corresponding UV-vis spectra, including a bathochromic shift (namely 8 nm) of the low-energy absorption (Fig. 2b).^27^ The restriction of intramolecular rotation of the phenyl rings within the stacks activates a radiative decay channel, as evidenced by the rapid increase in TPE emission upon cooling (Fig. 2c and d).

**Fig. 2:**
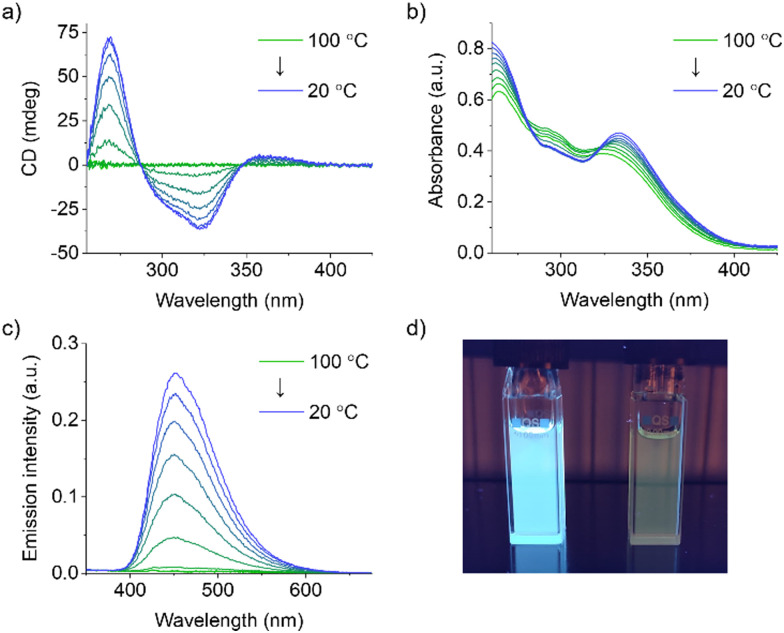
VT (a) UV-vis absorption, (b) CD and (c) fluorescence (700 V, *λ*_ex_ = 332 nm) spectra of 20 μM *S*-TPE solutions in decalin/TCE 9 : 1 (v/v) obtained by controlled cooling from 100 °C to 20 °C at a rate of 1 °C min^−1^ (optical path length: 10 mm). (d) Picture of 20 μM *S*-TPE solutions in decalin/TCE 9 : 1 v/v (left) and TCE (right) at 20 °C under 366 nm irradiation.

The self-assembly is likely driven by hydrogen-bond interactions between the amide functionalities, as indicated by the rupture of the supramolecular structures upon addition of an hydrogen-bond scavenger at the end of the cooling ramp (Fig. S8, ESI†). As such, a 0.4% volume fraction of MeOH is sufficient to cause the complete disappearance of the CD signal attributable to the supramolecular polymers. Stacking interactions between the monomeric units likely contribute to drive the assembly, with the latter being triggered in relatively apolar environments where the extended aromatic core experiences poor solvation. However, we speculate that the twisted conformation of the TPE core prevents extended π–π stacking between adjacent monomers in the assembly, ensuring a strong emissive character of the aggregates.

Although the non-sigmoidal shape of the CD cooling curves suggests a nucleation–elongation mechanism, simulations with a mass-balance model for cooperative polymerization do not provide an accurate fit in the nucleation phase (Fig. S9, ESI†).^28^ This is likely due to the coexistence of different aggregated species.^29^ Since TPE emission results from the restriction of intramolecular rotational modes, regardless of the nature of the aggregate, it also cannot serve as a readout to follow the supramolecular polymerization.

Atomic force microscopy (AFM) and emission microscopy experiments were performed on spin-coated and drop-casted samples prepared by deposition of 20 μM solutions of *S*-TPE in decalin/TCE 9 : 1 (v/v) on freshly cleaved mica or glass substrates, respectively. AFM studies revealed the formation of a network of tens of micrometers one-dimensional structures whose height profile (namely, 2 nm) corresponds to that expected for single fibers (Fig. 3a and Fig. S10, S11, ESI†). The formation of one-dimensional supramolecular structures was further demonstrated by emission spectroscopy, which revealed the highly fluorescent behavior of the assemblies (Fig. 3b and Fig. S12, ESI†).

**Fig. 3:**
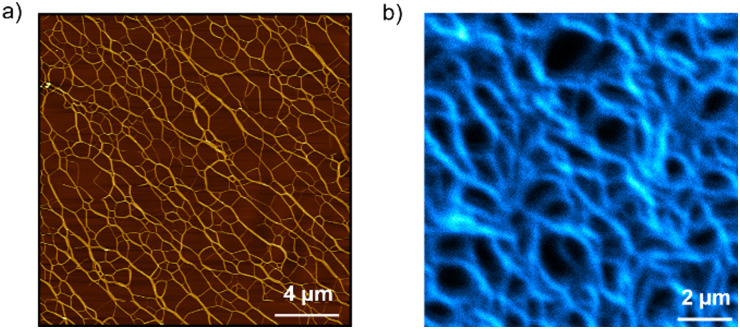
(a) AFM image of a spin-coated sample prepared by deposition of a 20 μM *S*-TPE solution in decalin/TCE 9 : 1 (v/v) on freshly cleaved mica. (b) Emission microscopy image of a drop-casted sample prepared by deposition of a 20 μM *S*-TPE solution in decalin/TCE 9 : 1 (v/v) on glass (*λ*_ex_ = 405 nm).

Overall, the results obtained from the study of the self-assembly of non-symmetric, propeller-shaped *S*-TPE monomer are consistent with the knowledge gained from the study of hydrogen-bonded supramolecular polymers made of *C*_3_-symmetric discotics.^20^

Next, we investigated the emissive properties of *S*-TPE supramolecular polymers embedded in optical nanocavities. The latter consist of dielectric nanoparticles arranged in a square lattice with a spacing close to the wavelength of visible light. These cavities exhibit high-quality optical resonances, known as Mie Surface Lattice Resonances (M-SLRs), resulting from the interaction between the localized Mie resonances of the individual nanoparticles and the in-plane diffraction orders (DOs) of the array.^30–35^ The M-SLRs concentrate the electromagnetic fields near the array surface. Consequently, when an equilibrated solution of supramolecular TPE assemblies is drop casted onto the nanocavity surface (Fig. S13, ESI†),^36^ the strong fields enhance the interaction of (the excitons in the) TPE fibers with light, resulting in enhanced emission at the resonant wavelengths of the cavity. These resonant wavelengths, which control the directional emission from the TPE fibers, can be tuned by modifying the cavity properties such as particle size, particle material, lattice symmetry, or lattice period.

In this connection, we designed an optical nanocavity consisting of TiO_2_ nanodisks with a diameter of 130 nm and a height of 94 nm on a quartz substrate (Fig. 4a), and varied the lattice period between 360 nm and 425 nm to control the emission spectrum of the TPE fibers on the cavity (Fig. 4c for SEM images and ESI† for fabrication details). The resonant energies of the in-plane diffraction orders of these arrays can be calculated using the 2D lattice equation:1
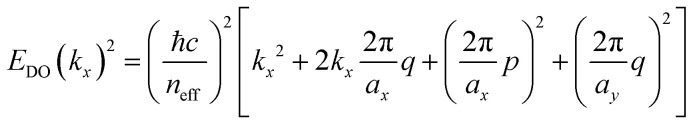
where *k*_*x*_ = *k*_0_ sin *θ* is the incident wave vector along the *x* direction, *n*_eff_ is the effective refractive index of the surrounding medium, (*p*, *q*) are the grating orders along the *x* and *y* directions and *a*_*x*_ and *a*_*y*_ are the lattice vectors along the *x* and *y* directions.

**Fig. 4:**
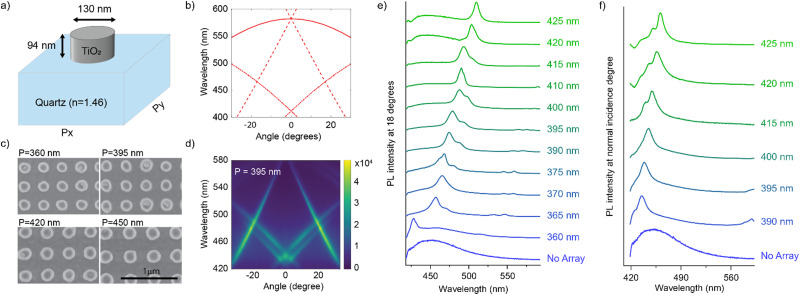
(a) Schematic representation of one unit cell of the nano-cavity. (b) Calculated energies of the in-plane diffraction orders for an array with *P*_*x*_ = *P*_*y*_ = 395 nm and *n*_eff_ = 1.47. The solid curve indicates the (±1, 0) orders, the dashed curve the (0, ±1), and the dash dotted curve the (±1, ±1) orders. (c) SEM images of the cavity for a period of 360, 395, 420 and 450 nm. (d) Angle dependent fluorescence intensity from the TPE fibers on top of the cavity with 395 nm period. (e) and (f) Normalized fluorescence intensity of the TPE-fibers on different arrays at an angle of (e) 18 degrees and (f) 0 degrees.

We plot the calculated (±1, 0), (0, ±1) and (±1, ±1) diffraction orders for an array with a lattice period of 395 nm in Fig. 4b. With TPE fibers placed on the array, angle-resolved emission is measured using a Fourier microscope (see ESI† for further details). The observed emission from the coupled system closely matches the dispersion of the calculated diffraction orders (Fig. 4d). Thus, the lattice design provides direct control over the emission spectrum of the TPE fibers on the array. It should be noted that due to the interaction of the DOs with the Mie resonances of the individual particles and excitons in the fibers, the actual resonances are slightly shifted with respect to the calculated DOs.^37^ The emission spectra at an angle of 18 degrees for different lattice constants (*a*_*x*_) are plotted in Fig. 4e, showing the tunability of the emission peak from 430 up to 510 nm. In Fig. 4f the emission in the normal direction is plotted, where the redistribution of fluorescence is controlled by the resonant wavelength (*λ*_res_) of the first diffraction order of the cavity. For these cases, eqn (1) simplifies to:2
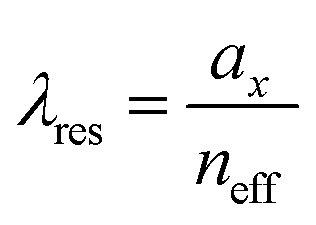


In summary, we have investigated the modulation of emission from H-type supramolecular polymers based on TPE chromophores by exploiting the surface lattice resonances of dielectric TiO_2_ nanoparticles. Spectroscopic studies in dilute decalin/TCE solutions revealed the hydrogen bonding-driven assembly of TPE-based monomers into one-dimensional assemblies with preferred helicity. Within the stacks, restriction of intramolecular rotation of the phenyl moieties allows emission. AFM and confocal microscopy revealed the formation of tens of micrometers long fibers. Implementation of the latter into optical nanocavities composed of TiO_2_ nanoparticle arrays resulted in a drastic redistribution of emission as a consequence of weak coupling to the M-SLR. The insights gained from this work pave the way for further studies on exciton-photon coupling in aggregated emissive systems and materials, opening new possibilities for the implementation of well-designed supramolecular assemblies in optoelectronics and photonics. The properties of H-type supramolecular polymers under different regimes of light–matter coupling and within chiral cavities will be investigated in the future in our laboratories.^38,39^

The authors acknowledge the support of the ICMS microscopy facilities, Dr Yuyang Wang for assistance with emission microscopy measurements, Stef Jansen for the computational modeling of supramolecular polymerization, Dr Stefan Meskers for fruitful discussion and the ICMS Animation Studio for the cartoon of the assembly. This work received funding from the European Research Council (H2020-EU.1.1., SYNMAT project, ID 788618), the Dutch Ministry of Education, Culture and Science for the Gravitation Program Functional Molecular Systems (024.001.035) and the Japan Society for the Promotion of Science for a Bilateral Joint Research Project (JPJSBP120239921). G. L. acknowledges a Marie Skłodowska-Curie Postdoctoral Individual Fellowship (101026072) for financial support. J. G. R. acknowledges the Nederlandse Organisatie voor Wetenschappelijk Onderzoek (NWO) for financial support through the Vici Grant No. 680-47-628.

## Conflicts of interest

There are no conflicts to declare.

## Supplementary Material

CC-060-D3CC05877H-s001
