# Supplementary material for: Tunable emission from H-type supramolecular polymers in optical nanocavities
Source: Chem Commun (Camb). 2024 Feb 9;60(20):2812–5. doi: 10.1039/d3cc05877h (PMC10913141; doi:10.1039/d3cc05877h)
Supplement: CC-060-D3CC05877H-s001 [file CC-060-D3CC05877H-s001.pdf]

Electronic Supplementary Material (ESI) for ChemComm.
This journal is © The Royal Society of Chemistry 2024

Supporting Information

Tunable emission from H-type supramolecular polymers in optical nanocavities

Giulia Lavarda,[a] Anton M. Berghuis,[b] Kripa Joseph,[a] Joost J. B. van der Tol,[a] Shunsuke Murai,[c] Jaime Gómez Rivas[b] and E. W. Meijer[a*]*

[a] Institute for Complex Molecular Systems and Laboratory of Macromolecular and Organic Chemistry, Eindhoven University of Technology, 5600 MB Eindhoven, The Netherlands.

[b] Eindhoven Hendrik Casimir Institute and Institute for Complex Molecular Systems, Eindhoven University of Technology, 5600 MB Eindhoven, The Netherlands.

[c] Department of Material Chemistry, Graduate School of Engineering, Kyoto University, Katsura, Nishikyo, 6158510 Kyoto, Japan.

* Correspondence to: g.lavarda@tue.nl, e.w.meijer@tue.nl

Table of Contents

1. Materials and methods S2
2. Synthesis and characterization of ***S*-TPE** S4
3. Self-assembly studies S8
    3.1 UV-vis absorption, circular dichroism and fluorescence spectroscopy S8
    3.2 Atomic force and emission microscopy S11
4. Dispersion of the arrays S15
5. References S16

1. Materials and methods

(1-Cyano-2-ethoxy-2-oxoethylidenaminooxy)dimethylamino-morpholino-carbenium hexafluorophosphate (COMU) and *N*,*N*-diisopropylethylamine (DIPEA) were purchased from Sigma-Aldrich. 4,4',4'',4'''-(Ethene-1,1,2,2-tetrayl)tetrabenzoic acid was purchased from A2B. Solvents used for synthesis were purchased from Biosolve B.V. Deuterated solvents were purchased from Cambridge Isotopes Laboratories. Decalin (mixture of *cis* and *trans*, anhydrous) and 1,1,2,2-tetrachloroethane (TCE) for spectroscopy were purchased from Thermo Fisher Scientific. All chemicals and solvents were used as received. The synthesis of (*S*)-3,7-dimethyloctylamine has already been reported.[1]

Thin layer chromatography (TLC) was carried out on aluminum sheets precoated with silica gel 60 F254 (Merck). Column chromatography was performed on Screening Devices silica gel (40-63 μm, 60 Å). ^{1}H and ^{13}C nuclear magnetic resonance (NMR) spectra were recorded on a Bruker ASCEND spectrometer. In ^{1}H-NMR and ^{13}C-NMR spectra, chemical shifts (δ) are expressed in ppm and are referenced to the residual peak of the solvent. Matrix-assisted laser desorption/ionization time of flight (MALDI-TOF) mass spectrometry (MS) was performed on a Bruker Autoflex Speed instrument. *Trans*-2-[3-(4-*tert*-butylphenyl)-2-methyl-2-propenylidene] malononitrile (DCTB) and cyano-4-hydroxycinnamic acid (CHCA) were used as matrices.

For all spectroscopic measurements, sealed Hellma Quartz Suprasil cuvettes with an optical path length of 1 cm or 0.1 cm were used. TCE solutions for spectroscopic measurements were prepared by weighing the desired amount of compound in a sealed vial and adding the required volume of solvent using Gilson MICROMAN positive-displacement pipettes to achieve the desired concentration. The solutions were then sonicated in a 40 °C water bath for 60 seconds and vortexed for 30 seconds. They were then transferred to sealed cuvettes. For the preparation of solutions in decalin/TCE mixtures, a stock solution in TCE was first prepared according to the procedure described above with a concentration 10 times higher than the target one. A volume of this solution was then transferred to another vial and the required volume of decalin was added to achieve the desired concentration. The resulting solutions were then vortexed for 30 seconds and stirred at 110 °C for 30 minutes. Subsequently, the samples were transferred while still hot into sealed cuvettes. All spectroscopic measurements were performed on freshly prepared solutions.

Steady-state absorption, circular dichroism (CD) and fluorescence spectroscopy were performed on a JASCO J-815 CD spectrometer equipped with a JASCO MPTC-490S temperature controller and a Jasco FMO-427S/15 emission monochromator. Variable temperature (VT) measurements were performed after heating the sample at 100 °C for 20 minutes in the spectrophotometer. A cooling rate of 1 °C/min was used for full spectra measurements at VT. The cooling ramp was stopped during the acquisition of the spectra. Single steady-state measurements at 20 °C were performed after heating the sample at 100 °C for 20 minutes followed by cooling to 20 °C at a rate of 1°C/min.

Atomic force microscopy (AFM) images were collected using a Cypher Environmental Scanner (ES) equipped with a closed cell and a normal laser diode. A heating and cooling stage was used to actively control the temperature at 20 °C. Silicon NCSTR probes (Oxford Instruments, spring constant k = 7.4 N/m; f = 160 kHz) with a tip height of 10-15 μm and a radius of 7 nm were used for all measurements. The AFM tip was thermally calibrated using the 'Get Real' function of the Igor Pro software. Subsequent

height images were acquired in repulsive tapping mode (phase <90) at a resolution of 1024x1024 pixels using a scan rate of ~2 Hz and an integral gain of 50-150. Image contrast was enhanced using first order planefit and flattening with Gwyddion v2.60. on. Emission microscopy images were acquired with a STED Abberior microscope in the confocal mode using a 405 nm laser (spectral range: 471-611 nm). Microscopy samples were prepared by either spin-coating or drop-casting 20 μM solutions of the compound in decalin/TCE 9:1 (v/v) onto freshly cleaved 1.5x1.5 cm sized mica (AFM) or 22x50 mm sized borosilicate glass (emission microscopy). Solutions for microscopy were prepared following the same procedure used for preparing solutions for spectroscopic measurements. Prior to deposition, the solutions were heated in the spectrophotometer at 100 °C for 20 minutes and then cooled to 20 °C at a rate of 1°C/min. A spin rate of 1000 rpm was used for the preparation of spin-coated samples.

The TiO_2 nanoparticle arrays were prepared on a SiO_2 glass substrate by electron-beam lithography followed by reactive ion etching. First, a TiO_2 thin layer with thickness of 94 nm was deposited on the substrate by RF (radio frequency) magnetron sputtering. Then, a resist (ZEP520A) was spin-coated and the nanohole array pattern was written by electron-beam lithography. After that, a Cr layer (120 nm) was deposited by electron-beam deposition, and the following lift-off process resulted in a Cr dot array pattern on the TiO_2 layer. Then, the TiO_2 layer was etched away by reactive ion etching with CHF_3 gas to make the nanoparticle array. The Cr dot mask was then removed by wet etching.

The energy and momentum resolved extinction of the arrays are measured in a Fourier microscope. The 40x, 0.6NA excitation objective (Nikon Plan Fluor) focuses a 400 nm laser onto the sample. The laser beam is generated by frequency doubling the output of a Ti:sapphire regenerative amplifier (Coherent Astrella), λ= 800 nm with a pulse duration of 150 fs. The fluorescence from the sample is collected by a 60x, 0.7 NA objective (Nikon Plan Fluor). The back focal plane of this objective is imaged on the spectrometer slit (Princeton Instruments SP2300) using 2 lenses in a 4f configuration. The spectrometer slit transmits the light along one of the principal axis of the array and disperses light on the CCD camera (Princeton Instruments ProEM:512) using a 150 lines/mm grating.

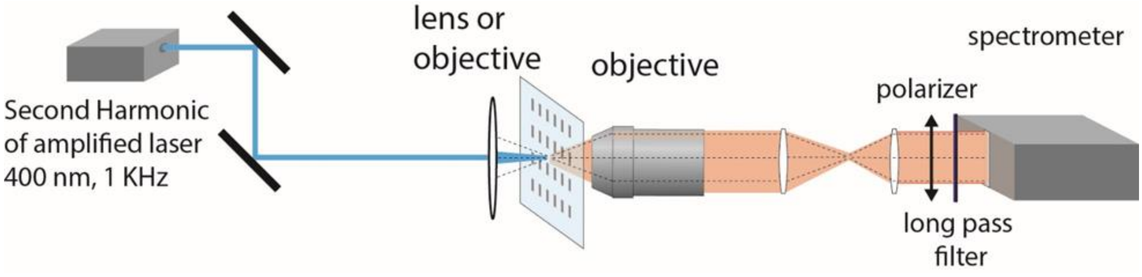

Figure S1. Schematic representation of Fourier microscope.

2. Synthesis and characterization of *S*-TPE

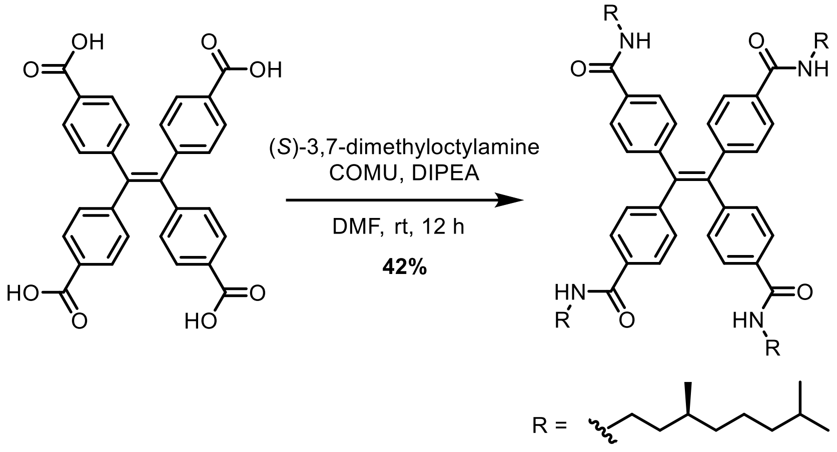

Scheme S1. Synthesis of ***S*-TPE** from the corresponding tetrabenzoic acid precursor.

4,4',4'',4'''-(Ethene-1,1,2,2-tetrayl)tetrabenzoic acid (150 mg, 0.30 mmol), COMU (632 mg, 1.48 mmol) and DIPEA (400 μL, 2.35 mmol) were dissolved in DMF (4 mL) in a 10 mL round bottom flask and the reaction mixture was stirred at room temperature for 15 min. At this point, (*S*)-3,7-dimethyloctylamine (232 mg, 1.48 mol) was added, and the mixture was stirred overnight at room temperature. The solvent was then evaporated under reduced pressure and the crude product was dissolved in ethyl acetate (15 mL). The solution was washed with aqueous HCl (1 M, 3x15 mL), saturated $NaHCO_3$ (3x15 mL) and water (3x15 mL). The organic phase was dried over $MgSO_4$, filtered, and evaporated to dryness under reduced pressure. The crude was purified by column chromatography using chloroform/methanol 95:5 (v/v) as eluent. Recrystallization from chloroform afforded ***S*-TPE** as a white solid in 42% yield (132 mg).

^{1}H-NMR (400 MHz, 3 vol% TFA-d_1 in chloroform-d_1): δ (ppm) = 7.47 (d, J = 8.4 Hz, 8H), 7.05 (d, J = 8.4 Hz, 8H), 6.41 (s, br), 3.52–3.39 (m, 8H), 1.67–1.57 (m, 4H), 1.55–1.38 (m, 12H), 1.34–1.22 (m, 12H), 1.20–1.09 (m, 12H), 0.92 (d, J = 6.5 Hz, 12H), 0.86 (d, J = 6.6 Hz, 24H). ^{13}C-NMR (101 MHz, 3 vol% TFA-d_1 in chloroform-d_1) : δ (ppm) = 169.63, 146.03, 141.45, 132.53, 131.64, 127.06, 39.31, 38.08, 37.15, 36.35, 30.91, 28.07, 24.73, 22.79, 22.69, 19.54. MS (MALDI-TOF): m/z calculated for $C_{70}H_{104}N_4O_4$: 1065.63; found: 1065.79 $[M+H]^+$.

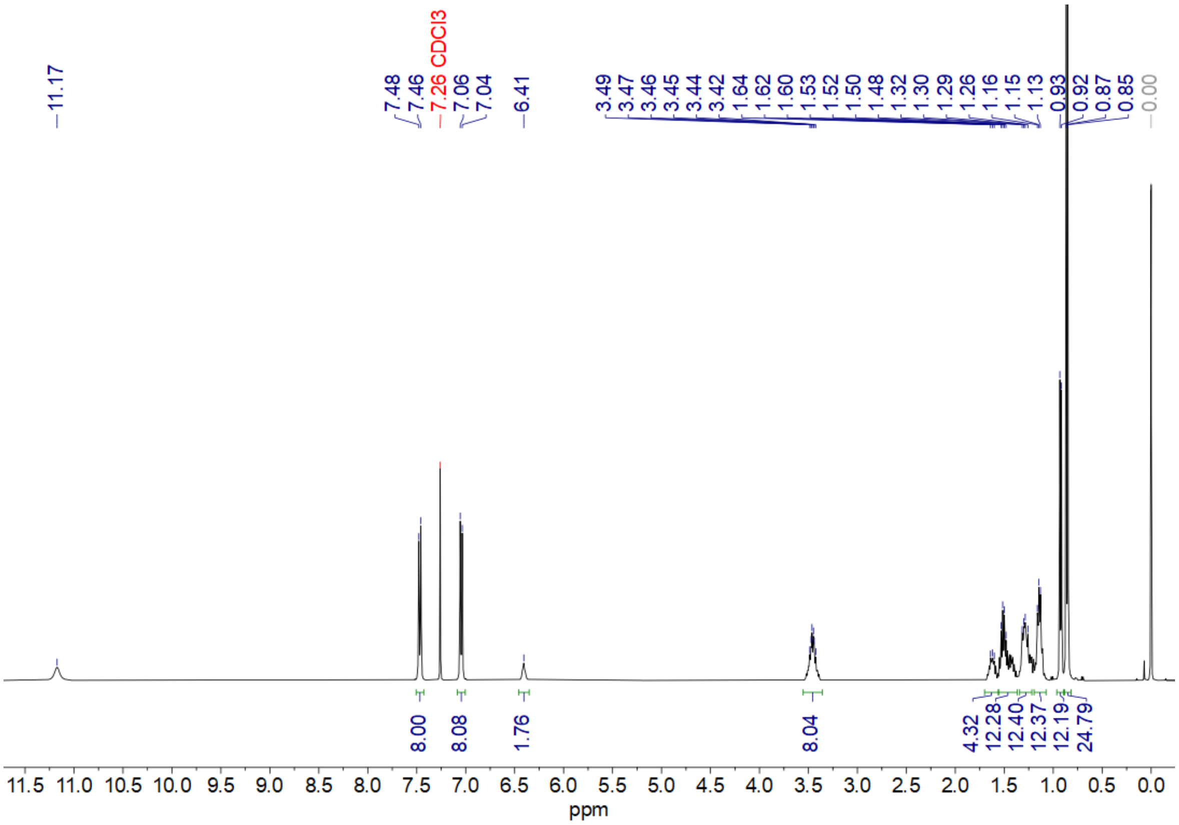

Figure S2. ^{1}H-NMR spectrum (400 MHz, 3 vol% TFA-d_1 in chloroform-d_1) of ***S*-TPE**.

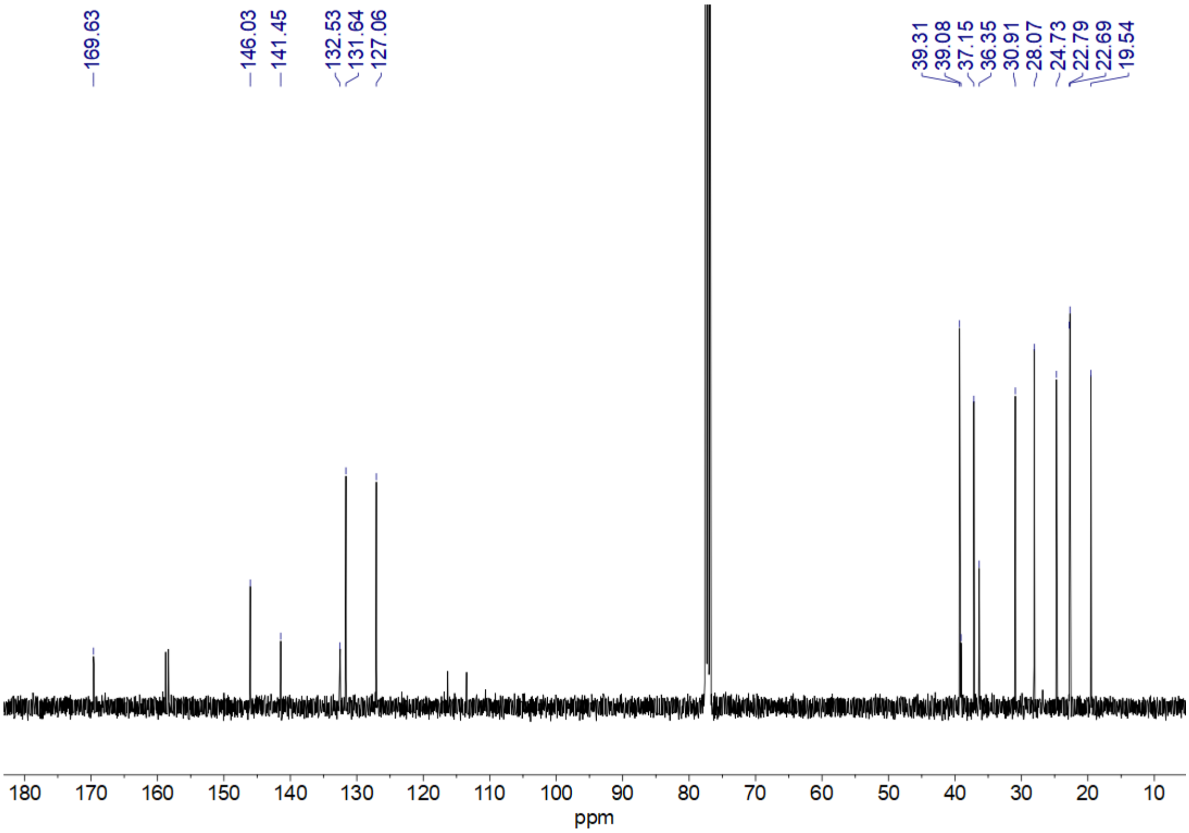

Figure S3. ^{13}C-NMR spectrum (400 MHz, 3 vol% TFA-d_1 in chloroform-d_1) of ***S*-TPE**.

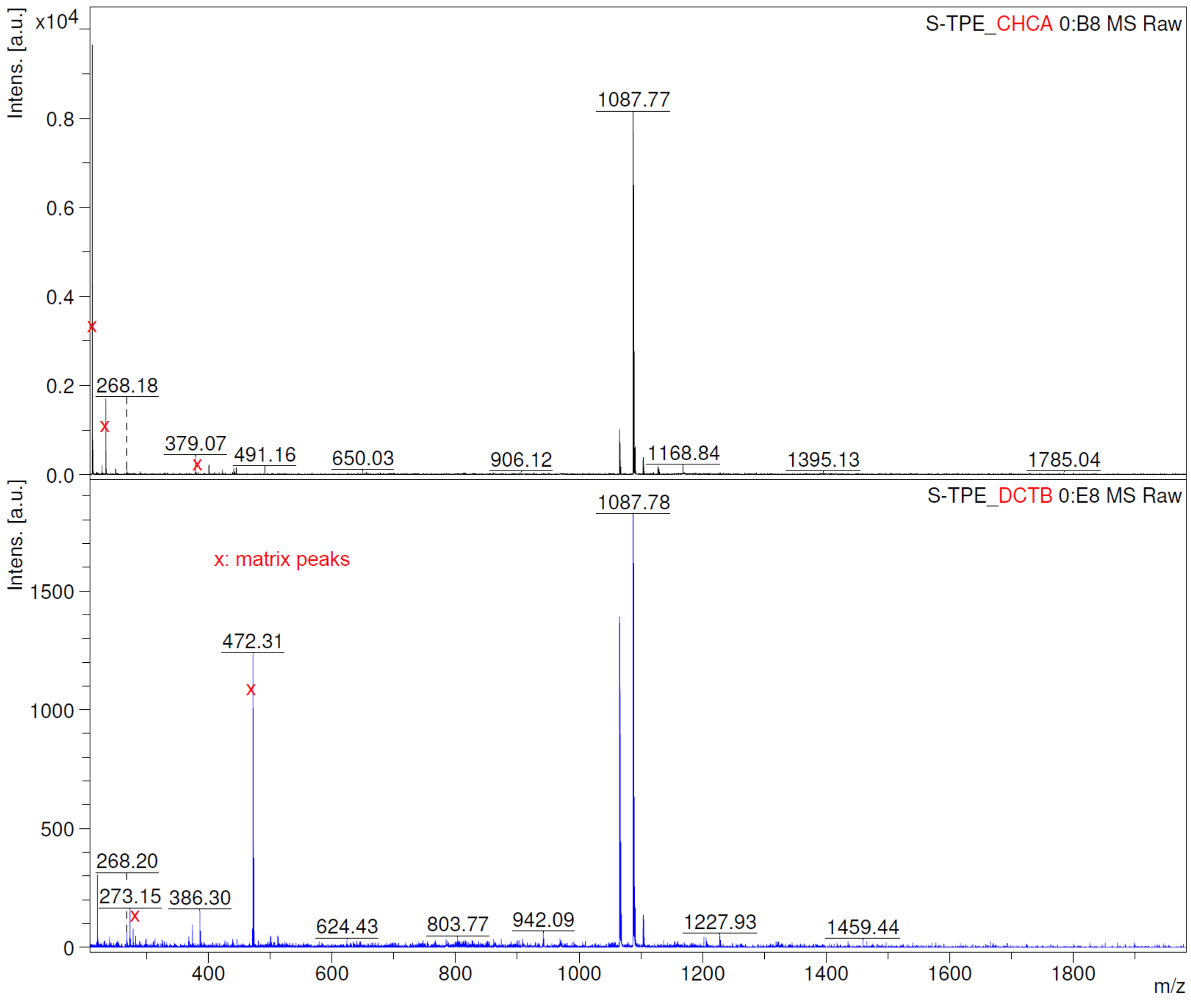

Figure S4. MALDI-TOF mass spectra (in CHCA and DCTB matrices) of ***S*-TPE**.

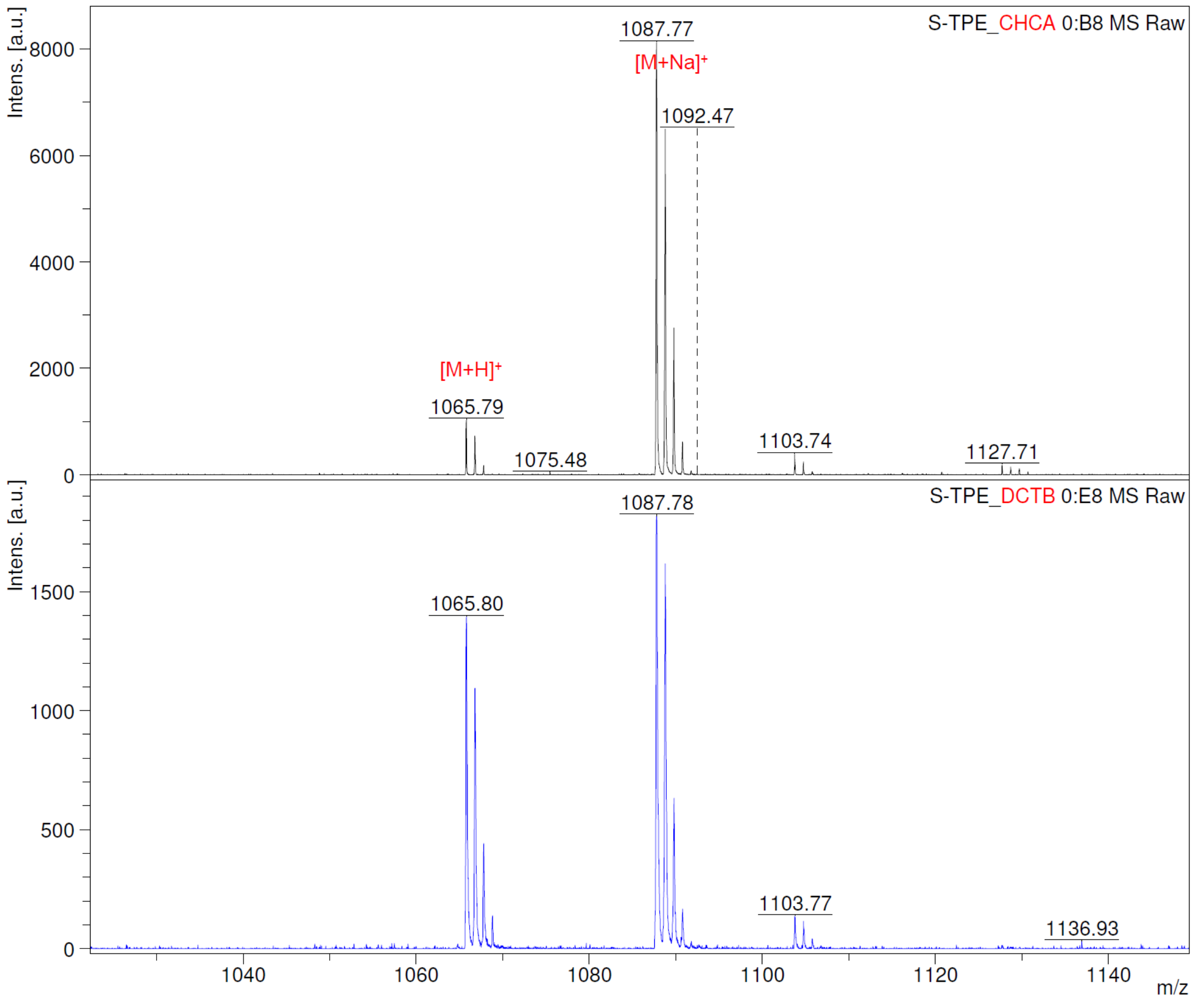

Figure S5. Zoom of MALDI-TOF mass spectra (in CHCA and DCTB matrices) of ***S*-TPE**.

3. Self-assembly studies

3.1 UV-vis absorption, circular dichroism and fluorescence spectroscopy

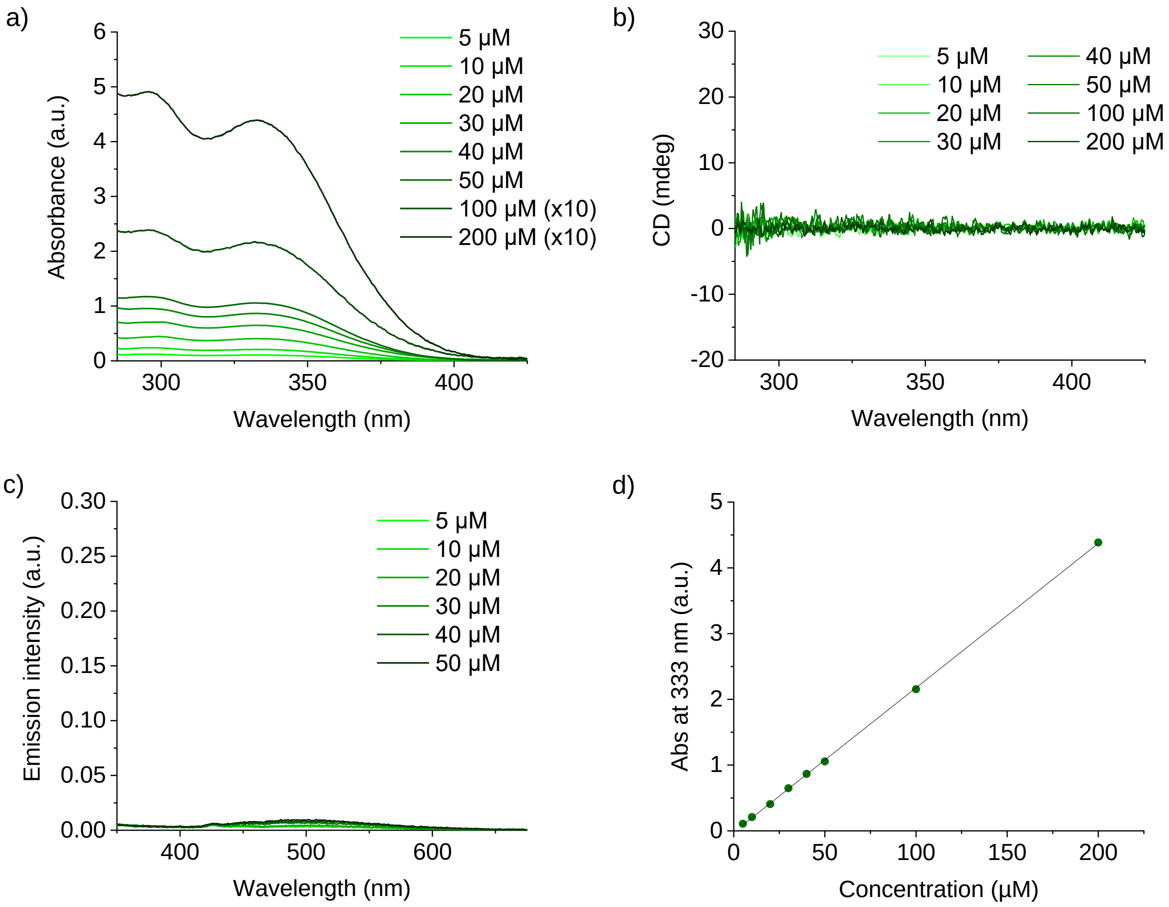

Figure S6. Concentration-dependent a) UV-vis absorption, b) CD and c) fluorescence (λ_{ex}=332 nm, 700 V) spectra of ***S*-TPE** solutions in TCE at 20 °C. Measurements were performed after heating the samples at 100 °C for 20 minutes and then cooling to 20 °C at a rate of 1°C/min. Optical path length: 10 mm for 5-50 μM solutions, 1 mm for 100-200 μM solutions. d) Linear correlation between absorbance at 333 nm and concentration. The absorbance values corresponding to the 100-200 μM solutions in (a) and (d) were multiplied by a factor of 10 to account for the difference in path length.

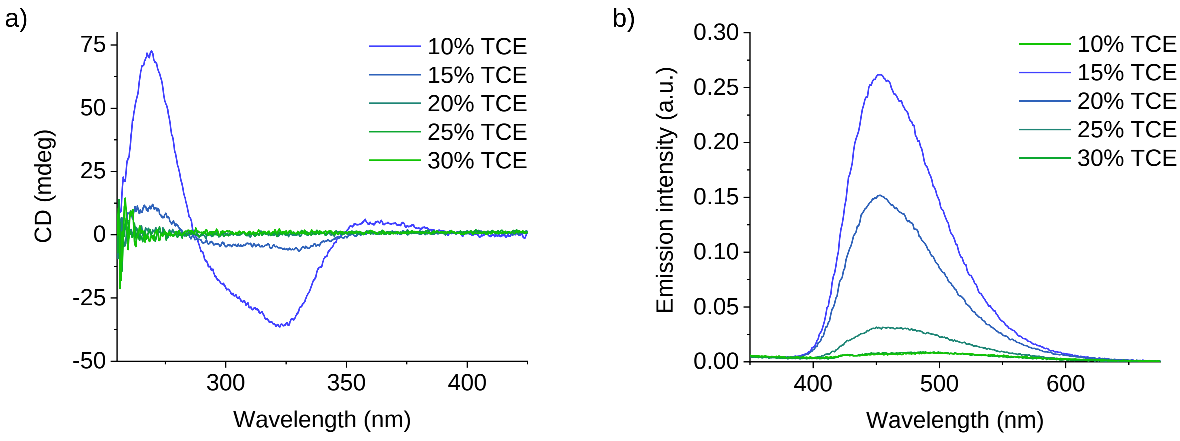

Figure S7. a) CD and b) fluorescence (λ_{ex}=332 nm, 700 V) spectra of 20 μM ***S*-TPE** solutions in decalin/TCE mixtures at 20 °C in the presence of different volume ratios of TCE. Measurements were performed after heating the samples at 100 °C for 20 minutes and then cooling to 20 °C at a rate of 1 °C/min. Optical path length: 10 mm.

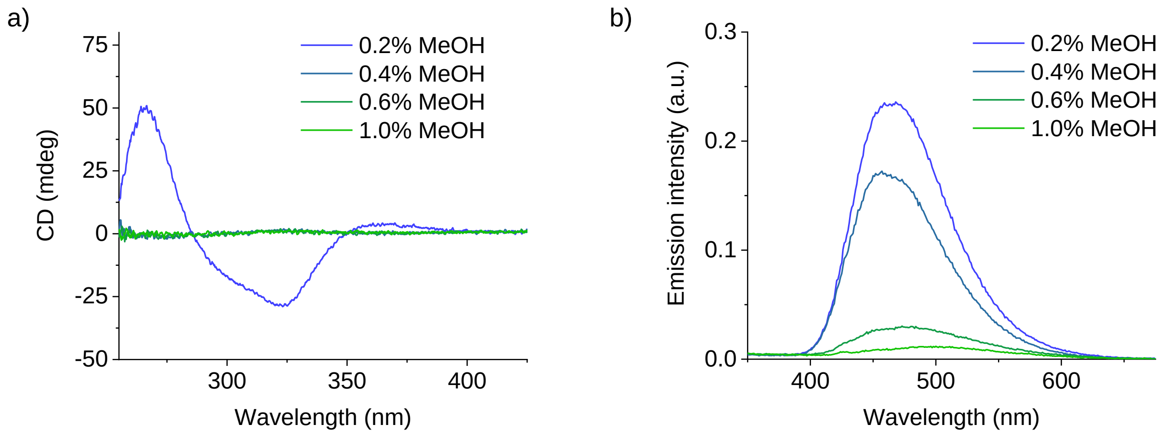

Figure S8. a) CD and b) fluorescence (λ_{ex}=332 nm, 700 V) spectra of 20 μM ***S*-TPE** in decalin/TCE 9:1 (v/v) at 20 °C after the addition of different volume ratios of MeOH as an H-bond scavenger. MeOH was added after heating the sample at 100 °C for 20 minutes and then cooling to 20 °C at a rate of 1°C/min. Optical path length: 10 mm.

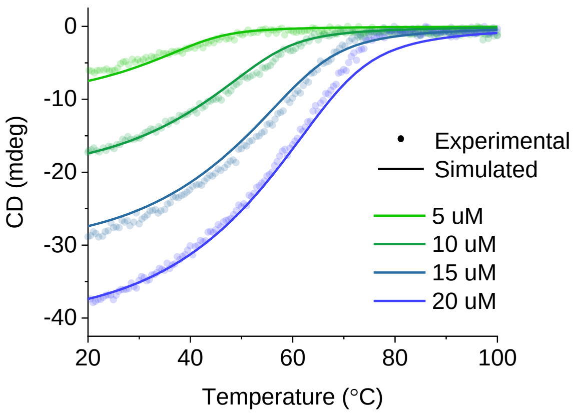

Figure S9. Experimental CD cooling curves (circles) of 5, 10, 15 and 20 μM ***S*-TPE** in decalin/TCE 9:1 (v/v) monitored at 322 nm obtained by controlled cooling from 100 °C to 20 °C at a rate of 1 °C/min (optical path length: 10 mm) and optimized fit of the mass-balance model for cooperative supramolecular polymerization[2] to the CD cooling curves (lines). Measurements were performed after heating the sample at 100 °C for 20 minutes. The thermodynamic parameters obtained from the fit are: ΔG_e = -47.7 kJ mol^{-1}; ΔS = -50.3 J mol^{-1} K^{-1}; NP (enthalpic nucleation penalty) = 10.3 kJ mol^{-1}.

3.2 Atomic force and emission microscopy

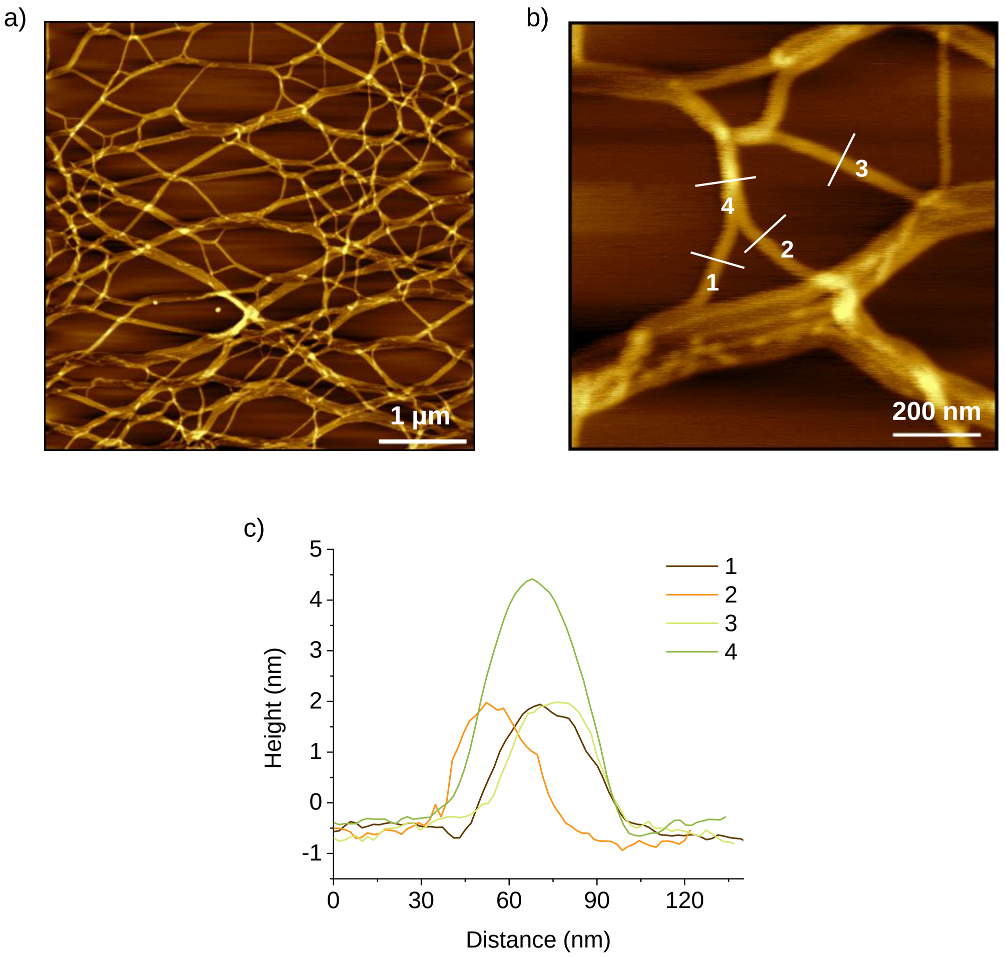

Figure S10. a,b) AFM images of drop-casted samples prepared by deposition of 20 µM ***S*-TPE** solutions in decalin/TCE 9:1 (v/v) on freshly cleaved mica. c) Height profile of ***S*-TPE** assemblies along the lines indicated in (b).

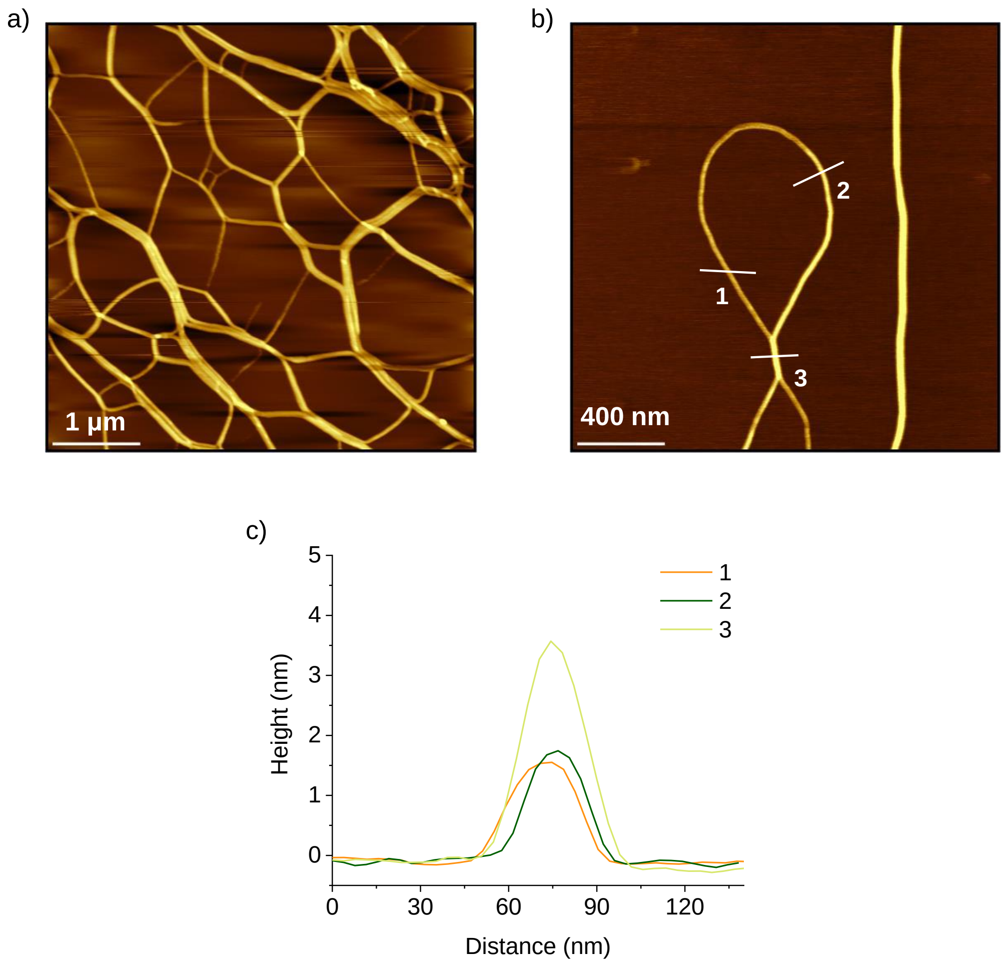

Figure S11. a,b) AFM images of spin-coated samples (spinning rate: 1000 rpm) prepared by deposition of 20 µM ***S*-TPE** solutions in decalin/TCE 9:1 (v/v) on freshly cleaved mica. c) Height profile of ***S*-TPE** assemblies along the lines indicated in (b).

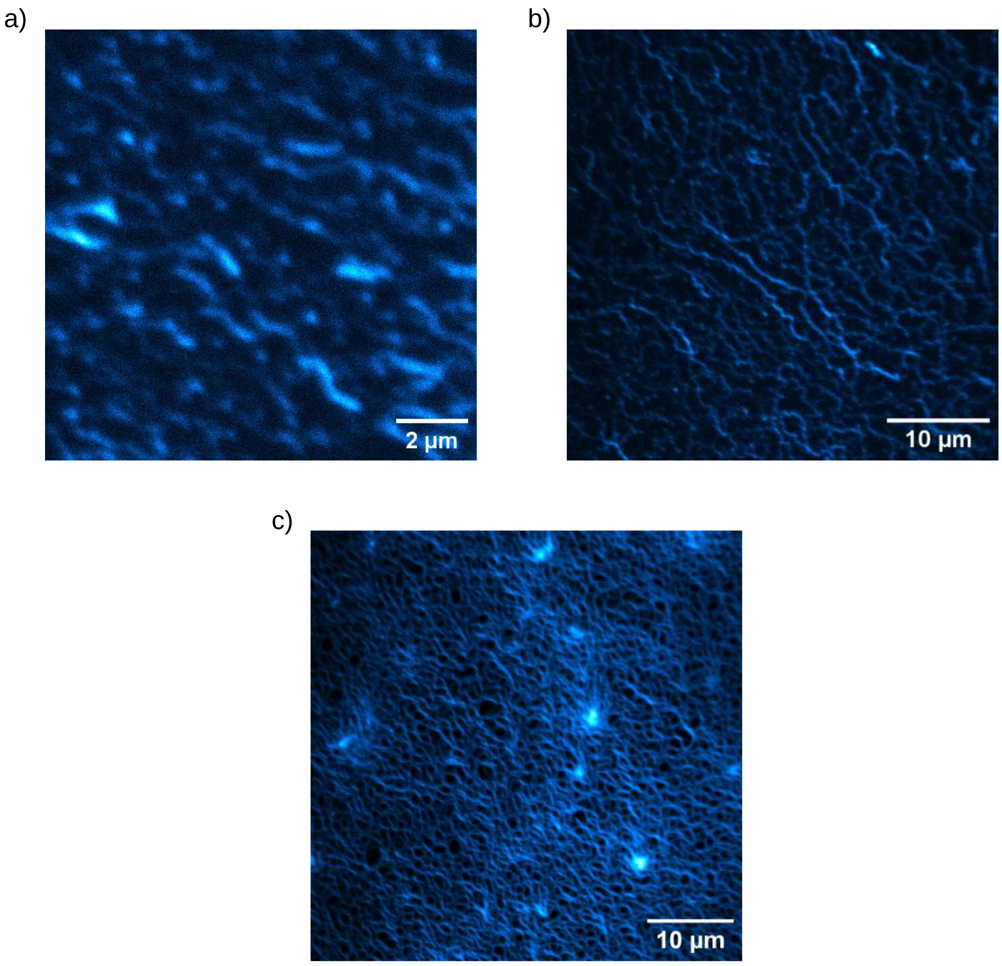

Figure S12. Emission microscopy images of a,b) spin-coated (spinning rate: 1000 rpm) and c) drop-casted samples prepared by deposition of 20 µM ***S*-TPE** solutions in decalin/TCE 9:1 (v/v) on borosilicate glass (λ_{ex} = 405 nm).

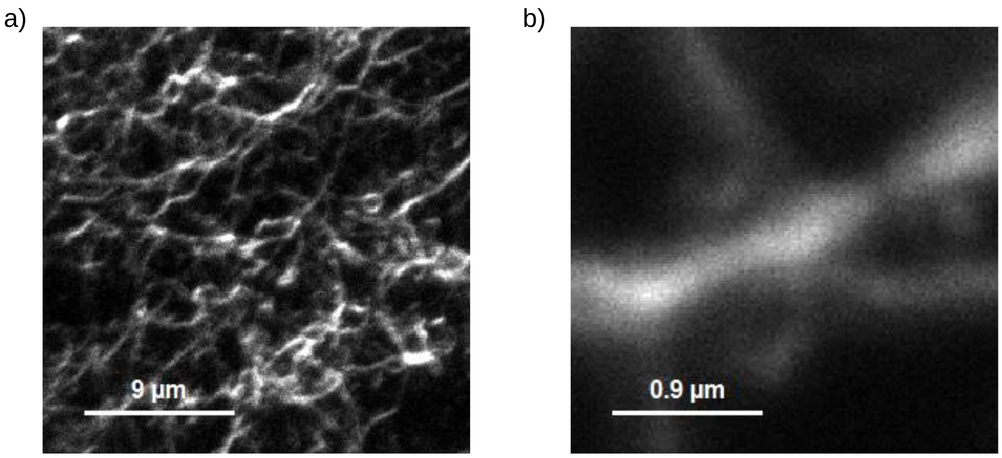

Figure S13. Confocal microscopy images of drop-casted samples prepared by deposition of 20 µM ***S*-TPE** solutions in decalin/TCE 9:1 (v/v) on the TiO_2 nanoparticle arrays.

4. Dispersion of the arrays

The angle dependent photoluminescence from the ***S*-TPE** fibers on top of the nanocavities is plotted below. With increasing lattice period, a red shift of the emission is visible. The width of the cavity modes differs slightly between the arrays, which is mainly attributed to small differences in the nanoparticle dimensions. On the top right of each panel, the maximum number of counts of each image is given. The overall emission from fibers on top of the array is higher than outside the array, but there is a large variation in intensity due to the uneven coverage of the arrays with the *S*-TPE fibers. The arrays with a period of 380 and 385 nm were not covered and therefore these dispersions are missing.

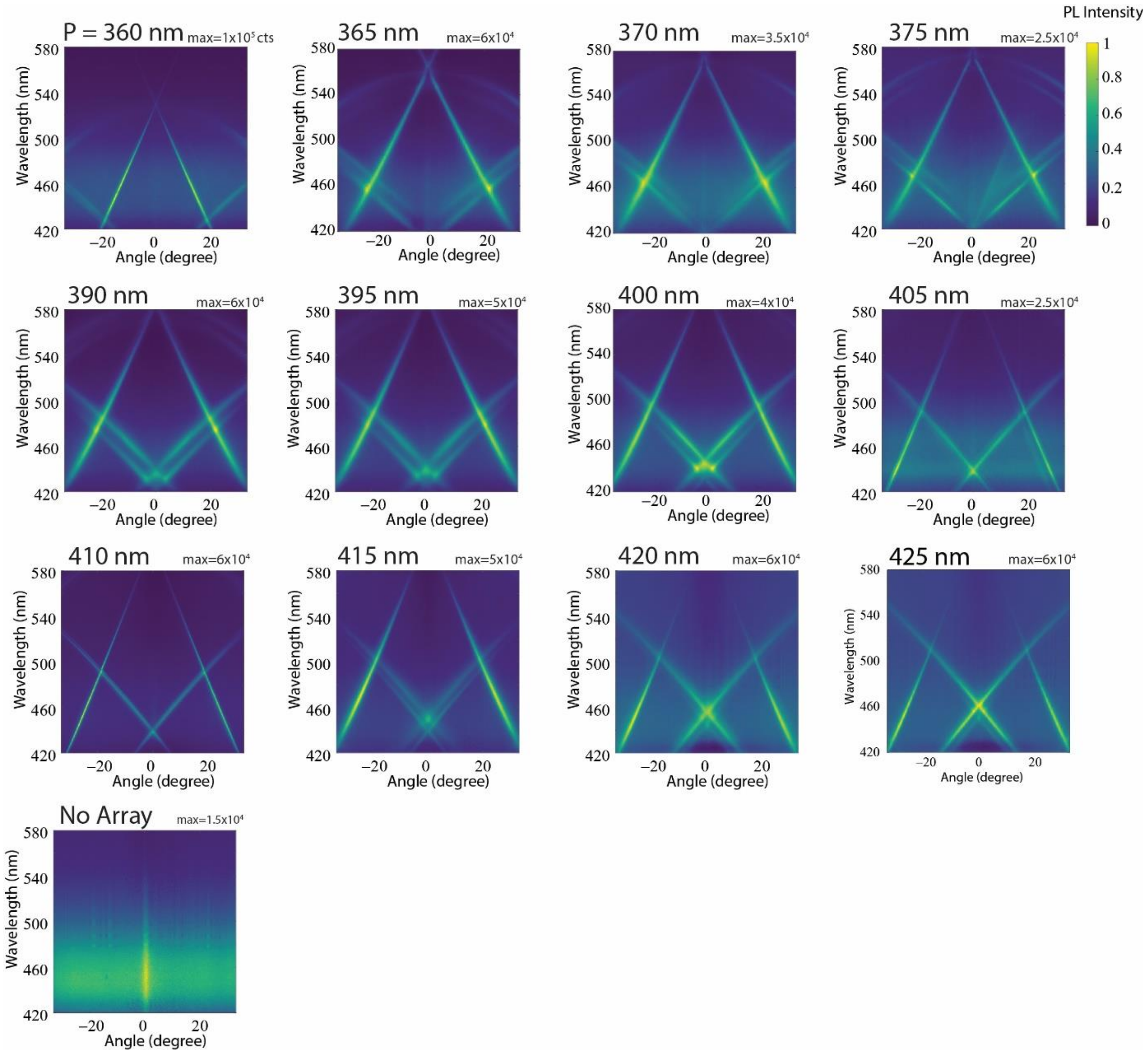

Figure S14. Angle dependent fluorescence maps of the TiO_2 nanoparticle arrays covered with ***S*-TPE**.

5. References

[1] S. Cantekin, T. F. A. de Greef, A. R. A. Palmans, *Chem. Soc. Rev.* 2012, **41**, 6125.

[2] D. H. Zhao, J. S. Moore, *Org. Biomol. Chem.* 2003, **1**, 3471.
